# The Assessment of Ankle Range-of-Motion and Its Relationship with Overall Muscle Strength in a Cross-Section of Soccer Players

**DOI:** 10.3390/sports11010012

**Published:** 2023-01-05

**Authors:** Piergiorgio Francia, Carlo Ferri Marini, Leonardo Bocchi, Barbara Piccini, Giuseppe Seghieri, Ario Federici, Sonia Toni, Francesco Lucertini

**Affiliations:** 1 Department of Biomolecular Sciences—Division of Exercise and Health Sciences, University of Urbino, 61029 Urbino, Italy; 2 Department of Information Engineering, University of Florence, 50139 Florence, Italy; 3 Meyer Children’s Hospital, 50139 Florence, Italy; 4 Epidemiology Unit, Agenzia Regionale Sanità, 50141 Florence, Italy

**Keywords:** footballers, flexibility, sport practice, muscle strength, hand grip, injuries

## Abstract

Soccer (football) practice can induce a limitation of ankle range of motion (ROM) that is a possible risk factor for injury and other negative consequences over time. The main objective of this research was to investigate the effects of soccer practice on ankle ROM throughout the entire period of a sports career of soccer players (SP). Furthermore, the relationship between ankle ROM and muscle strength in SP of different ages was studied. A total of 204 SP (range 6.7–45.1 years) and 87 controls (range: 7.5–45.2 years) matched for age, body mass index (BMI), and gender, were assessed. Ankle ROM in both plantar flexion (APF) and dorsiflexion (ADF) in addition to handgrip strength (HGS) were evaluated using an inclinometer and the Jamar hydraulic hand dynamometer, respectively. The comparison between SP and control groups showed a significant reduction in ankle ROM of both APF (26.3 ± 7.2° vs. 32.6 ± 7.4°; d = −0.90; *p* < 0.001) and ADF (95.5 ± 15.6° vs. 105.5 ± 15.8°; d = −0.66; *p* < 0.001). In SP, the results of the ANOVAs test indicate that age had a significant effect on ADF (F = 4.352, *p* = 0.038, partial eta-squared (η_p_^2^) = 0.015) but not on APF (F = 0.430, *p* = 0.746, η_p_^2^ = 0.001). Moreover, considering only the SP, a weak inverse correlation between ADF and HGS group ADF was found (r_s_ = −0.27; *p* < 0.001). Factors such as the non-linear trend of growth in young SP could hinder the definition of the relationship between ankle ROM, age, and muscle strength. However, the appropriate consideration of age and muscle strength could facilitate the management of ankle ROM in PF of different ages.

## 1. Introduction

Soccer (football) is one of the most practiced team sports in many countries worldwide. In particular, the majority of young people as well as a large number of adults who participate in amateur leagues choose to play soccer [1,2].

The very widespread practice of soccer entails the necessity of studying its effects on soccer players. Playing soccer is important because it is a significant source of physical activity and can have many positive health effects on both young people and adults [3,4]. However, practicing soccer continuously has also been associated with some negative consequences [3,5] such as those affecting the ankle (talocrural) joint [6,7,8].

Among them, several studies published over the last 50 years suggest that soccer players (SP) may show an alteration of ankle range of motion (ROM) [9,10,11,12]. A deficit of ankle ROM can cause several negative consequences. In this sense, it is known that a limited ankle ROM could represent a risk factor and be a predictor [13,14] of injuries such as ankle sprains [7,15,16] and overuse condition [17,18,19]. These negative consequences may be due to the adverse effects that a limited ankle ROM may have on the biomechanics of movements such as: walking, running, and jumping [16,20,21,22,23]. In addition, it has been suggested that static-dynamic balance [8,24,25] as well as posture [26,27,28,29] may also be negatively affected by the presence of limited ankle ROM. Consequently, the study of such alterations is recommended in order to provide appropriate prevention and treatment [14,15,26,30].

The causes of reduced ankle ROM in SP are still partially unknown. However, an important role has been attributed to repeated injuries of the joint [7,15,20]. All ankle ligaments and joint capsule in addition to other periarticular structures can be subjected to injuries and alterations due to sport practice [6,7,31]. In particular, injuries of the ankle in SP can be either traumatic or resulting from repeated sports-specific microtrauma. The role of repeated microtrauma is considered important because it is common among SP and it can lead to a condition of overuse of the joint and periarticular structures [7,14,24,31]. In this sense, it is known that physical contact between players and the repeatedly kicking a soccer ball can cause micro-trauma [8,32,33]. Moreover, sport specific and high intensity movements such as jumping or running with frequent and rapid changes of speed and direction which generate torque forces may lead to non-contact ankle ligament injury [6,8,32]. These types of injuries can result in a partial deafferentation from articular and periarticular structures with possible negative consequences for the balance, posture, and movements of SP [8,28,29,34,35].

Analysis of the causal factors and consequences of limited ankle ROM in SP indicate how complex vicious cycles might develop in these players. Vicious cycles can also be supported by alterations in muscle activity that SP can often have. As such, since early studies regarding the relationship between soccer practice and ankle ROM, it has been hypothesized that muscle activity may also play a role in limiting ankle joint ROM. In particular, some authors have hypothesized that SP can instinctively seek greater ankle joint stability in order to protect the ankle from continuous micro-traumatic or traumatic events [10,32,36]. This stability of the ankle could be ensured by an increased co-contraction of the agonist and antagonist muscles involved in joint movements [37,38,39]. In this sense, the muscle strength level and tightness of the leg muscles can affect this sport-specific adaptation and ankle ROM [13,15,40]. Moreover, over time the practice of soccer can cause an important increase in the stiffness of the periarticular soft tissues and muscle-tendon structures [7,32,37,41]. This adaptation can also play a role in increasing the stability of the ankle and in reducing ankle ROM. All of this highlights the typical role of muscle activity for the movement of the ankle in SP. In particular, muscle activity is important both to ensure sport specific movement and to protect the ankle joint itself. Consequently, the study of the relationship between ankle ROM and hand grip strength (HGS) is important in order to better understand the causes of the limited ankle ROM itself. Moreover, it is also important to consider the result of a muscle tendon imbalance caused by rapid growth or changes in training level [41,42].

This is very interesting considering that limited ankle ROM, in addition to being very evident in adults, can also be present in young SP with a short history of sports practice [11,12,32,43]. Regarding the assessment of muscle strength, it is known that the HGS test provides a well-established and objective score to the point that it is considered important for following subjects throughout their growth [44,45]. Moreover, the HGS test is easily and quickly available, including in a sports environment, it is indicative for generalized muscle strength and allows a comparison with reference values [46,47,48,49]. In this sense, the study of the relationship between ankle ROM and HGS is important in order to better understand the causes of the limited ankle ROM itself.

The stiffness of the ankle joint in SP would paradoxically be at the same time, first a preventive factor for ankle injuries and then a risk factor for the same injuries. All this indicates that analyzing the effects of practicing soccer on ankle ROM and its relationship with muscle strength could provide useful information on actions aimed at the prevention of ankle ROM alterations and its possible immediate or long-term negative consequences. The main objective of this research was to investigate the effects of soccer practice on ankle ROM throughout the entire period of a sports career of SP. Furthermore, the relationship between ankle ROM and muscle strength in SP of different ages was studied.

## 2. Methods

A total of 291 subjects, 204 soccer players (SP) [range: 6.7–45.1 years] and 87 controls [range: 7.5–45.2 years] matched for age, gender, and BMI were evaluated in this study (Table 1). Ankle (or talocrural) ROM in both plantar and dorsiflexion in addition to hand grip strength were examined. Detailed characteristics of the study participants are shown in Table 1.

**Figure 1:**
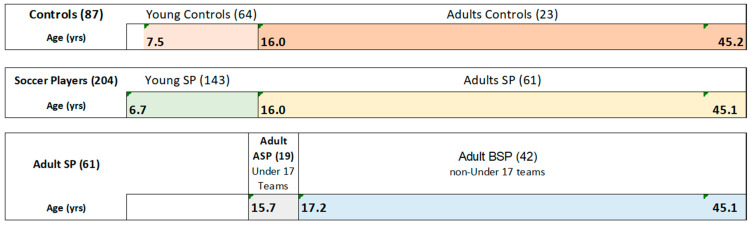
Range of age for groups: soccer players (204), controls (87), under 17 teams, and non-under 17 teams (19). Abbreviations: SP: soccer players; Adult ASP: under 17 team group; Adult BSP: adult non-under 17 teams groups.

The control group consisted of sedentary subjects or subjects who practiced sports for which no significant effects on ankle ROM are known such as basketball, volleyball, athletics [9,50,51]. Regarding adult, we decided to evaluate two senior male soccer teams because they represent the large part of SP [1,2]. For an appropriate analysis of the effects of growth on ankle ROM, SP and controls were assigned to two different groups: young (<16 years) and adult (>16 years). The age of 16 was chosen because previous studies showed that in adolescence ankle ROM can decrease significantly [32]. Two subjects aged 15.9 and 15.7 were included in the adult group because they trained and played with a team participating in the under 17 championship (Figure 1).

In order to better study the effect of growth on ankle ROM, the group of SP adults was also divided into adults A SP (under 17 teams) and adults B SP (non-under 17 teams; Figure 1). Participants’ age, height, weight, the dominant leg used for kicking, the sport performed, the number of weekly training sessions, and the years of sport practice were recorded. Moreover, the type of footwear used, the playing surface where the training sessions took place, the duration of the training sessions, the injuries suffered in addition to the job activity of SP were considered [12,15]. Body mass index (BMI) was calculated as the ratio between body weight in kilograms and height in squared meters (kg/m^2^).

The physical examination included foot inspection for the presence of deformity, injuries, and traumas that might affect ankle ROM. Exclusion criteria were: age less than 6 years or greater than 50, presence of diabetes, other diseases, as well as orthopedic and/or surgical complications at baseline that can affect ankle ROM, and for the soccer players, soccer practice for less than 6 months continuously. The measurements were taken at least one month after the start of the championship and immediately before the first weekly training session. All participants, parents or legal guardians were informed of the purpose of the study and its experimental procedures before obtaining their written informed consent and the enrolment in the study. The study protocol and the consent forms were approved by the Pediatrics Ethics Committee of Meyer Children’s Hospital in Florence (protocol number: 161/2016 on 29 September 2016) and by Ethics Committee of University of Urbino Carlo Bo (cod. CESU20221118VER37 November 2020). The study was carried out according to the principles expressed in the Declaration of Helsinki.

### 2.1. Determination of Joint Range of Motion

**Figure 2:**
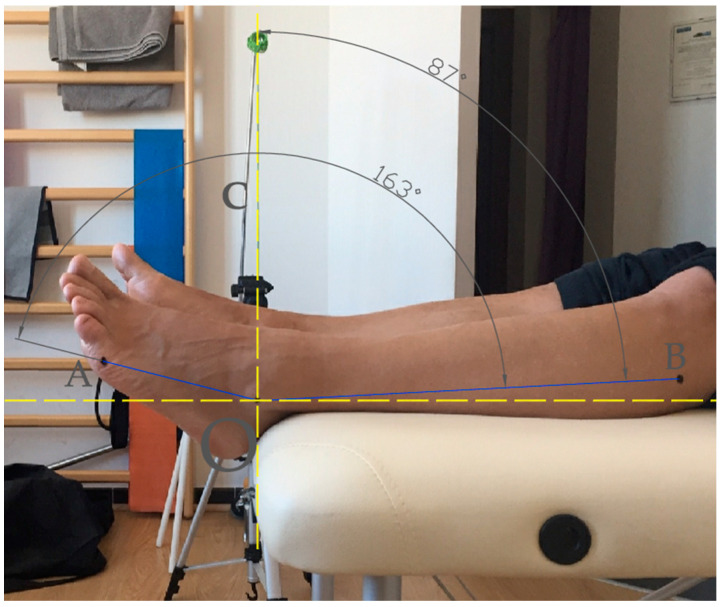
Ankle joint active range of motion assessment on the sagittal plane.

Ankle (or talocrural) joint ROM was assessed as described in previous studies [12,50,52]. Briefly, an inclinometer was used to measure the active ROM of the ankle joint in plantar flexion (APF) and dorsiflexion (ADF), see Figure 2 for a visual representation.

Players were instructed to lie supine on a fixed treatment table with their feet crossed and ankles resting in line with the edge. The ipsilateral knee was placed on a rigid support that was 5 cm high. 

After marking the fifth metatarsal bone with a dermographic pen, the inclinometer (Fabrication Enterprises Inc., White Plains, New York, USA) was placed along the diaphysis of the bone, with one extremity on the distal condyle. The ankle joint was in its natural resting position on the sagittal plane, with the subtalar joint in a neutral position. The maximal active APF and ADF angles were measured and reported as the means of three consecutive readings. The total ankle ROM (ATOT) was the sum of APF and ADF. This method yields a limited mean standard deviation (SD) of three consecutive readings of the ankle ROM (1.1 ± 0.9 degrees of plantar flexion and 1.4 ± 1.1 degrees of dorsiflexion) [53]. The same operator, who had more than 10 years of experience, measured ankle ROM. The dominant lower limb was identified by asking the players which lower limb they preferred to use to kick a ball with. The operator who assessed ankle ROM was not aware of the participants’ dominant limb [54].

Ankle joint active range of motion was measured as the changes in the ankle angle from the starting resting position (set at 0° in the inclinometer) to the maximal active plantar flexion and dorsiflexion positions (the total range of motion was the sum of plantar flexion and dorsiflexion). The ankle joint position on the sagittal plane was measured as the angle with the vertex at the center of the lateral malleolus (O) and straight lines passing through the head of the fifth metatarsal bone (A) and the second through the center of the head of the fibula (B).

### 2.2. Determination of Hand Grip Strength

Hand grip strength was assessed using a Jamar hydraulic hand dynamometer (model 5030J1). The dominant upper limb was determined by asking the players which hand they used to write with [45]. The examiner provided standardized explanations and demonstrated the posture to be maintained, how to hold the dynamometer, and how to perform the test before its beginning. Specifically, participants’ hand grip strength was tested in the standing position, with the arms by the side of the body, shoulder adducted in a neutral position, and elbow flexed at 90° with the forearm parallel to the ground and pronated to maintain the display of the dynamometer on the frontal plane [55]. Only the dominant hand was tested. Participants performed a trial to become familiar with the device. Then, the participants were instructed to maintain the same posture and grip on the dynamometer handle and to squeeze the dynamometer as hard as possible for three seconds without moving the rest of their body three times. The highest force applied was automatically recorded by the peak-hold needle. The test was interrupted if there was any pain present while it was being performed. During the test, no verbal encouragement was given and the examiner counted the seconds and provided the signal to stop. Three tests were performed consecutively with a scheduled 15-sec rest period between the trials. The average value of the three attempts was reported [45,48]. The adjustable dynamometer handle was placed in the second grip position (i.e., 4.76 cm), while for younger subjects, it could be moved, if needed, to the first position (i.e., 3.5 cm) to allow them to properly handle the device with adequate provision for the fingers. The device calibration was assured by using a new tool and verifying that, in the absence of load, the needle position was “zero”. Additionally, the device was placed on a rigid surface, and the accuracy of the measurements provided by the device’s needle at different loads was verified by applying 10 kg and 20 kg weights. The Jamar hydraulic hand dynamometer, which is widely used in clinical practice, has been used in this research since it is a well-validated tool for measuring the maximum isometric muscle strength of the hand. Additionally, this dynamometer has high reproducibility and test-retest and inter-rater reliability in children and adults [44,55,56,57,58].

### 2.3. Statistical Analysis

Data were reported as mean ± SD. Range of motion values was expressed in degrees (°). Three separate two-way ANOVAs were performed to assess the effects of age and group on ADF, APF, and ATOT. Cohen’s *d* was also computed and considered small (*d* = 0.2), medium (*d* = 0.5), and large (*d* = 0.8) effect sizes based on the benchmarks suggested by Cohen (1988) [59]. A statistical normality test was performed using Shapiro–Wilk tests. The comparisons between the two groups were carried out using the independent *t*-test or the nonparametric test: (*) Mann–Whitney. The association between the ankle ROM and AGE, BMI, and hand strength was also evaluated in controls and separately in young and adult SP as well as considering all subjects assessed using Pearson or Spearman’s correlation coefficients. Since age, body mass index, and muscle strength had a role in the reduction of ankle ROM, multiple linear regression analysis was carried out considering ADF, APF, and ATOT (expressed in degrees) as dependent variables and age, BMI, and hand strength as independent variables in adult SP or in young SP. Additional models were also evaluated using a backward elimination procedure in order to maximize the prediction ability of the models. The analyses were performed using Stata (StataCorp, v.13, StataCorp, College Station, TX, USA) and SPSS Statistics (IBM, v.25, IBM Corp, Armonk, NY, USA) software. The α level of statistical significance was set at 0.05.

## 3. Results

Soccer player and control groups had a similar distribution among young and adults (data not shown). The groups of volleyball and basketball players have instead shown an ankle ROM in line with what has been reported in large-size samples studies that show how ankle ROM in plantar and dorsiflexion is about 70° [51,60,61,62]. Regarding the two-way factorial ANOVAs test, the assumption of equality of variance (Levene’s test) of ADF and ATOT across groups was not met. For this reason, ADF and ATOT values were transformed using a base-10 logarithm before performing the test. For SP, the results of the ANOVAs indicate that age had a significant effect on ADF (F = 4.352, *p* = 0.038, partial eta-squared (η_p_^2^) = 0.015) but not in APF (F = 0.430, *p* = 0.746, η_p_^2^ = 0.001) and ATOT (F = 0.264, *p* = 0.095, η_p_^2^ = 0.009). The comparison between the groups showed a significant reduction in ankle range of motion of both APF and ADF in soccer players compared to controls (*p* < 0.001; Table 1). A similar result was obtained by considering young and adult soccer players and controls separately (Table 2).

From the comparison between the adult and young groups, ankle ROM was significantly higher in young SP compared to the adult SP (*p* < 0.001; Table 2). Conversely, ankle ROM was similar in adult controls compared to young controls (138.9 ± 20.5° vs. 137.8 ± 13.9°; Table 2). Regarding the group of adult SP, from the comparison of the under 17 teams (adults APF) with those of the senior soccer division (adults BSP) only the ADF was significantly lower in adults BSP (*p* < 0.006; Table 3; Figure 1).

Considering all SP investigated (Table 3), ATOT and ADF were weakly inversely correlated with Age (r_s_ = −0.17; *p* = 0.015; r_s_ = −0.20; *p* < 0.005; respectively) and HGS (r_s_ = −0.22; *p* = 0.002; r_s_ = −0.27; *p* < 0.001; respectively; Table 4).

The multiple linear regression analysis performed did not show any significant effects of age, BMI, and hand grip strength on ankle ROM (Table 5).

## 4. Discussion

The trend of the ankle joint range of motion of soccer players for the whole sport lifespan, from children up to adults, was investigated in this study. Moreover, the relationship between ankle ROM and muscle strength was considered. The comparison between SP and controls shows how the practice of soccer induces a significant reduction in ankle ROM (Table 1). This difference was also confirmed by considering young subjects and adults separately (Table 2). The presence of a limited ankle range of motion in soccer players (SP) seems paradoxical considering that soccer requires the best possible ability to control the ball with the feet while a limited ankle ROM may adversely affect this capacity [26,32,63]. In particular, results from ANOVAs test confirmed how age has a significant effect on ankle dorsiflexion but not on plantar flexion. The marked reduction of ankle plantar flexion already detected in young SP when compared with young controls (Table 2) could anticipate and hide the effect of age on ankle plantar flexion itself.

In order to better investigate the effect of the age on ankle ROM, further analyses were carried out considering young people (<16 years) and adults (over 16 years) separately. In particular, the comparisons between adult and young groups highlighted how the ankle ROM was significantly higher in young SP compared to the adult SP. Similar values of ankle ROM were instead found considering young and adult control groups (Table 2). Further analyses were performed by investigating the effect of the age on ankle ROM of SP, comparisons were carried out by dividing adult SP group into under 17 teams (adults APF) and non-under 17 teams (adults BSP; Table 3; Figure 1). Interestingly, this comparison highlighted how the ankle ROM is already strongly reduced in the SP of the under 17 teams and the ankle dorsiflexion was lower than that of the adult BSP group (Table 3). However, in the interpretation of this result it is important to consider how the players of the under 17 team trained one more time a week than adult SP of the senior soccer division (3 vs. 2) and used a different playing field (grass vs. synthetic). These factors could justify, at least in part, the ankle ROM values found in the adult APF and adults BSP groups. As a whole, these results indicate how the effects of soccer practice on ankle ROM occur precociously in children and they are almost fully present in late adolescence.

The analysis of the relationship between ankle ROM (dorsiflexion and plantarflexion) and the age or muscle strength of the PF showed only weak correlations. This result could be, at least in part, due to the non-linear trend of the ankle ROM reduction in SP. Among the causes of this non-linear trend, the changes related to the development of young SP and in particular the somatic and physiological ones could play an important role. In this sense, it is known that the growth of young SP and in particular puberty, may result in evident effects on the musculotendinous structures. In addition, it may be important to consider how the trend of development of muscle strength is not fully superimposable to that of bone tissue and height [41,64,65]. While children show a tendentially linear growth of muscle strength at the onset of pubertal growth spurt, approximately at 11–12 years for boys, [66,67,68] the skeletal growth typically occurs before musculotendinous growth [41,64]. In fact, the peak height velocity is about a year before peak growth velocity of body mass [41,65]. This time lag can determine a period in which the same muscle strength, and in particular the leg muscles, may be less effective in stabilizing the ankle while promoting its ROM in SP. Subsequently, the increased muscle strength may lead to an increased stabilizing action capacity although it can negatively affect the ankle ROM. In this sense, it is known that the increase in muscle-tendon stiffness reaches adult levels by late adolescence (16–18 years old) [64,69,70]. In fact, starting from 13–14 years of age, there is an acceleration of the pace with which muscle strength increases with an evident spurt during adolescence [41,67,70,71,72].

Overall, the results of this study suggest the usefulness of considering both the age and the muscle strength trend in monitoring the ankle ROM of SP. However, these results are only partially in agreement with those reported in some previous studies. As for senior male SP, almost forty years ago, Hattori and Ohta (1987) measured the range of motion of the ankle joint in 68 male soccer players (18–22 years) and in 66 non-athletic male student controls. The results of the study showed a significant difference in ankle ROM between the two groups. This difference was about 20 degrees considering plantar and dorsiflexion together [32]. This difference is similar to that found in our study (Table 1). Regarding the effect of growth on ankle ROM, in 2019 Cejudo et al. evaluated ankle dorsiflexion ROM in 72 young SP of 8–19 years. In particular, it was reported that despite having found higher mean values in younger subjects (under 10 years), no fully significant differences were found among SP of different ages. However, the ankle ROM showed a lower value passing from the under 10 players to the under 12 players, re-increasing in the under 14 SP and then decreasing again in the under 19 players [73]. The results for a larger sample of soccer players were published even more recently by the same authors. In this study, Robles-Palazón and colleagues (2022) studied the joint range of motion of the ankle and other joints of the lower limb in 286 male soccer players (age range: 10–19 years). The study results showed no significant changes in ankle ROM in players of different ages [43]. This result appears in contrast to those of our study. However, regarding the studies of Cejudo and Robles-Palazón it is important to consider that only dorsiflexion of the ankle was considered. This may, at least in part, explain the differences in the results obtained.

This study has limitations to consider. The study was carried out considering a sample of male subjects only without considering professional soccer players and the players positions. Furthermore, increasing the number of subjects investigated both for adult SP and for young SP could favor the description of the relationship between ankle ROM and soccer practice in footballers of different ages. Even if the BMI was assessed, the study did not consider the level of development of the subjects investigated. The use of an upper extremity test such as the HGS test can be considered a limit with respect to the study of the relationship between ankle ROM and muscle strength in SP. However, it is important to consider that HGS test is indicative of generalized muscle strength and it can be performed easily and quickly even in a sports environment [46,47,48,49]. Furthermore, this test is widely used in sports and clinical settings, provides a well-established and objective score [44,45,46,74]. All this can contribute to favoring the use of the HGS test for the evaluation of the SP and its relationship with the ankle ROM.

The results of this study relating to the significant reduction of ankle ROM, the ankle ROM values in SP of different ages, and the relationship between ankle ROM and muscle strength, suggest the need to organize activities aimed at the appropriate management of the ankle ROM in SP. This could have a positive effect on the prevention of injuries and other acute and chronic consequences associated with the presence of reduced ankle ROM. At the same time, further studies aimed at better describing the relationship between ankle ROM and age or muscle strength in SP and involving a larger number of subjects appear necessary.

## 5. Conclusions

With this study, data relating to ankle ROM were obtained for the entire period of a soccer career. In particular, the results achieved highlighted how male soccer players (SP) may show a significant reduction in ankle joint range of motion. This alteration may also be present in children from the first years of sport activity. Although the reduction in ankle ROM is already significant in young SP, ankle stiffness worsens significantly in adult SP. In apparent contrast to that statement, the ankle joint range of motion values measured in the SP of the under 17 teams were similar or lower than those of the SP of amateur leagues. Factors such as the non-linear trend of somatic growth could explain some of this apparent discrepancy. In particular, during puberty ankle ROM could be less affected by the practice of soccer as a consequence of the effects of the same puberty on the somatic growth. At the end of this period, ankle ROM could rapidly deteriorate as a result of the maturation of the musculotendinous and joint structures. This condition could also explain the weak correlation found between ankle ROM and age of muscle strength of SP. Furthermore, this relationship could also be affected by the marked early effect of soccer on the ankle plantar flexion. This condition could anticipate and hide the effect of age on the ankle plantar flexion itself. The need to prevent injuries and maximize the positive health effects of sports practice raises the importance of studying the relationship between ankle ROM and muscle strength in SP of different ages. In this sense, further studies involving a greater number of subjects appear necessary to better describe this relationship.

## Figures and Tables

**Table 1:** Main characteristic, ankle range of motion, and muscle strength of all soccer players and controls.

	All SP (n = 204)	All Controls (n = 87)	*p* Value	Cohen’s *d*
Age (years)	15.5 ± 7.5	15.9 ± 9.9	0.962 *	−0.06
BMI (kg/m^2^)	19.9 ± 2.9	20.1 ± 3.7	0.427 *	−0.16
HGS (kg)	27.8 ± 12.9	27.9 ± 11.4	0.182 *	−0.10
APF (degrees)	26.3 ± 7.2	32.6 ± 7.4	**<0.001**	−0.90
ADF (degrees)	95.5 ± 15.6	105.5 ± 15.8	<0.001 *	−0.66
ATOT (degrees)	121.8 ± 18.0	138.1 ± 19.2	<0.001 *	−0.92
∆ R-L (degrees)	5.3 ± 4.6	5.6 ± 5.0	0.655 *	−0.08

Note: Values are mean ± SD. Comparisons among groups were performed using *t*-test for independent samples or Mann–Whitney (*) test. Abbreviations: APF: ankle plantar flexion; ADF: ankle dorsiflexion; ATOT: total ankle range of motion; BMI: body mass index; SP: soccer players; HGS: handgrip strength; Δ: difference; R-L: right-left.

**Table 2:** Main characteristic, ankle range of motion and muscle strength of young and adult soccer players and controls.

	Adult SP (n = 61)	Adult Controls (n = 23)	*p* Value	Cohen’s *d*	Young SP (n = 143)	Young Controls (n = 64)	*p* Value	Cohen’s *d*
Age (years)	25.2 ± 6.9	28.0 ± 8.7	0.204 *	−0.39	11.3 ± 1.9	11.6 ± 1.8	0.594 *	0.17
BMI (kg/m^2^)	22.5 ± 2.1	23.4 ± 2.3	0.108	−0.41	18.6 ± 2.4	19.3 ± 3.6	0.380 *	0.26
HGS (kg)	44.2 ± 7.5	47.5 ± 8.8	0.125	−0.43	20.7 ± 6.7	23.8 ± 7.3	0.005 *	0.46
APF (degrees)	26.0 ± 5.7	33.5 ± 7.0	**<0.001**	−1.23	26.4 ± 7.8	32.5 ± 7.7	**<0.001**	0.79
ADF (degrees)	89.0 ± 10.5 ^	104.2 ± 13.1	**<0.001**	−1.36	98.3 ± 16.6 ^	106.4 ± 16.5	0.003 *	0.49
ATOT (degrees)	115.0 ± 12.9 ^	137.8 ± 13.9	<0.001 *	−1.73	124.7 ± 19.1 ^	138.9 ± 20.5	<0.001 *	0.73
∆ R-L (degrees)	4.4 ± 3.5	5.4 ± 5.2	0.252 *	−0.41	5.7 ± 4.9	5.6 ± 5.0	0.789 *	−0.03

Note: Values are mean ± SD. Comparisons among groups were performed using *t*-test for independent samples or Mann-Whitney (*) test; ^, significant difference (*p* < 0.001) between adult and young SP groups. Abbreviations: APF: ankle plantar flexion; ADF: ankle dorsiflexion; ATOT: total ankle range of motion; BMI: body mass index; SP: soccer players; HGS: handgrip strength; Δ: difference; R-L: right-left.

**Table 3:** Main characteristic, ankle range of motion and muscle strength of adult APF (under 17 teams) and adult BSP (non-under 17 teams) groups.

	Adult APF (n = 19) (Under 17 Teams)	Adult BSP (n = 42) (Non-Under 17 Teams)	*p*-Value	Cohen’s *d*
Age (years)	16.6 ± 0.4	29.0 ± 4.5	-	
BMI (kg/m^2^)	21.2 ± 1.5	23.1 ± 2.0	**<0.001**	−0.99
HGS (kg)	42.9 ± 5.9	44.7 ± 8.1	0.381	−0.24
APF (degrees)	27.1 ± 6.3	25.5 ± 5.5	0.322	0.28
ADF (degrees)	83.6 ± 9.0	91.4 ± 10.3	**0.006**	−0.79
ATOT (degrees)	110.7 ± 12.6	116.9 ± 12.7	0.131 *	0.49

Note: Values are mean ± SD. Comparisons among groups were performed using *t*-test for independent samples or Mann–Whitney (*) test. Abbreviations: APF: ankle plantar flexion; ADF: ankle dorsiflexion; ATOT: total ankle range of motion; BMI: body mass index; HGS: handgrip strength.

**Table 4:** Correlation matrix for soccer players and controls between ankle plantar flexion, ankle dorsiflexion, total ankle range of motion and age, BMI, and HGS.

.	APF		ADF		ATOT	
	r_s_	*p*-Value	r_s_	*p*-Value	r_s_	*p*-Value
**SP (204)**						
Age	0.03	0.674	−0.20	**0.005**	−0.17	**0.015**
BMI	−0.16	**0.029**	−0.29	**<0.001**	−0.30	**<0.001**
HGS	0.05	0.452	−0.27	**<0.001**	−0.22	**0.002**
**Controls (87)**						
Age	−0.11	0.328	−0.12	0.282	−0.16	0.129
BMI	−0.11	0.333	−0.18	0.100	−0.22	**0.039**
HGS	−0.05	0.667	−0.12	0.282	−0.15	0.196

Note: Comparisons were performed using Spearman’s rho test (rs). Abbreviations: APF: ankle plantar flexion; ADF: ankle dorsiflexion; ATOT: total ankle range of motion; BMI: body mass index; SP: soccer players; HGS: handgrip strength.

**Table 5:** Multiple linear regression analysis considering ankle dorsiflexion (ADF), ankle plantar flexion (APF), and total ankle range of motion (ATOT) (expressed as degrees) as dependent variables and age, body mass index (BMI), and hand strength (HGS) as independent variables in adult or in young soccer players (SP).

	Adult SP (61)			Young SP (143)		
	ß-Reg. Coef.	*p*-Value	*p*-Model	ß-Reg. Coef.	*p*-Value	*p*-Model
**APF**						
			0.312			0.266
Intercept	36.383	<0.001		34.326	<0.001	
BMI	−0.585	0.162		−0.558	0.091	
AGE	−0.074	0.523		−0.076	0.903	
HGS	0.103	0.332		0.170	0.288	
**ADF**						
			0.102			0.469
Intercept	72.649	<0.001		115.736	<0.0001	
BMI	−0.126	0.864		−0.974	0.131	
AGE	0.427	**0.042**		−0.138	0.910	
HGS	0.197	0.296		0.150	0.631	
**ATOT**						
			0.348			0.158
Intercept	109.032	<0.001		150.062	<0.0001	
BMI	−0.712	0.451		−1.531	**0.034**	
AGE	0.352	0.184		−0.215	0.875	
HGS	0.300	0.215		0.320	0.360	

Note: ß-Reg. Coef.: unstandardized coefficients.

## Data Availability

The data presented in this study are available on reasonable request from the corresponding author.

## List of Abbreviations

Adult APF	Players of Under 17 Teams
Adult BSP	Adult players non-Under 17 Teams
APF	Ankle Plantar Flexion
ADF	Ankle Dorsiflexion
ATOT	Total Ankle Joint Mobility
BMI	Body Mass Index
SP	Soccer Players
HGS	Hand Strength
R-L	Right-Left
ROM	Range of Motion
r_s_	Spearman’s rho

## References

[1] FIFA Communications Division (2007). FIFA Big Count 2006; Statistical Summary Report. https://digitalhub.fifa.com/m/55621f9fdc8ea7b4/original/mzid0qmguixkcmruvema-pdf.pdf.

[2] Study Center FIGC (2017). Report Calcio 2017. https://www.pwc.com/it/it/publications/assets/docs/reportcalcio-2017.pdf.

[3] Merkel D.L. (2013). Youth sport: Positive and negative impact on young athletes. Open Access. J. Sport. Med..

[4] Krustrup P., Krustrup B.R. (2018). Football is medicine: It is time for patients to play!. Br. J. Sport. Med..

[5] Bergeron M.F., Mountjoy M., Armstrong N., Chia M., Côté J., Emery C.A., Faigenbaum A., Hall G., Kriemler S., Léglise M. (2015). International Olympic Committee consensus statement on youth athletic development. Br. J. Sport. Med..

[6] Armenis E., Pefanis N., Tsiganos G., Karagounis P., Baltopoulos P. (2011). Osteoarthritis of the ankle and foot complex in former Greek soccer players. Foot Ankle Spec..

[7] Golanó P., Dalmau-Pastor M., Vega J., Batista J.P., d’Hooghe P., Kerkhoffs G. (2014). Anatomy of the Ankle. The Ankle in Football. Sports and Traumatology.

[8] Read P.J., Oliver J.L., De Ste Croix M.B., Myer G.D., Lloyd R.S. (2016). Neuromuscular Risk Factors for Knee and Ankle Ligament Injuries in Male Youth Soccer Players. Sports Med..

[9] Travers P.R., Evans G.P. (1976). Annotation limitation of mobility in major joints of 231 sportsmen. Br. J. Sport. Med..

[10] Ekstrand J., Gillquist J. (1982). The frequency of muscle tightness and injuries in soccer players. Am. J. Sport. Med..

[11] Rein S., Fabian T., Weindel S., Schneiders W., Zwipp H. (2011). The influence of playing level on functional ankle stability in soccer players. Arch. Orthop. Trauma Surg..

[12] Francia P., Ferri Marini C., Toni S., Mencarelli A., Iannone G., Lucertini F., Brandoni G., Monteiro-Soares M., Federici A., Piccini B. (2020). The effect of an adapted training protocol on ankle joint mobility of young soccer players. Med. Sport..

[13] de Noronha M., Refshauge K.M., Herbert R.D., Kilbreath S.L., Hertel J. (2006). Do voluntary strength, proprioception, range of motion, or postural sway predict occurrence of lateral ankle sprain?. Br. J. Sport. Med..

[14] Terada M., Pietrosimone B.G., Gribble P.A. (2013). Therapeutic interventions for increasing ankle dorsiflexion after ankle sprain: A systematic review. J. Athl. Train..

[15] Kaumeyer G., Malone T.R. (1980). Ankle injuries: Anatomical and biomechanical considerations necessary for the development of an injury prevention program. J. Orthop. Sports Phys. Ther..

[16] Mason-Mackay A.R., Whatman C., Reid D. (2017). The effect of reduced ankle dorsiflexion on lower extremity mechanics during landing: A systematic review. J. Sci. Med. Sport.

[17] Kaufman K.R., Brodine S.K., Shaffer R.A., Johnson C.W., Cullison T.R. (1999). The effect of foot structure and range of motion on musculoskeletal overuse injuries. Am. J. Sports Med..

[18] Sahillioglu A., Cerrahoglu L. (2021). The relationship of the foot and ankle structure with overuse injuries in licensed footballers: A prospective cohort study. J. Sports Med. Phys. Fit..

[19] Mahieu N.N., Witvrouw E., Stevens V., Van Tiggelen D., Roget P. (2006). Intrinsic risk factors for the development of achilles tendon overuse injury: A prospective study. Am. J. Sports Med..

[20] Brockett C.L., Chapman G.J. (2016). Biomechanics of the ankle. Orthop. Trauma.

[21] You J.Y., Lee H.M., Luo H.J., Leu C.C., Cheng P.G., Wu S.K. (2009). Gastrocnemius tightness on joint angle and work of lower extremity during gait. Clin. Biomech..

[22] Drewes L.K., McKeon P.O., Kerrigan D.C., Hertel J. (2009). Dorsiflexion deficit during jogging with chronic ankle instability. J. Sci. Med. Sport.

[23] Aronow M.S., Diaz-Doran V., Sullivan R.J., Adams D.J. (2006). The effect of triceps surae contracture force on plantar foot pressure distribution. Foot Ankle Int..

[24] Basnett C.R., Hanish M.J., Wheeler T.J., Miriovsky D.J., Danielson E.L., Barr J.B., Grindstaff T.L. (2013). Ankle dorsiflexion range of motion influences dynamic balance in individuals with chronic ankle instability. Int. J. Sports Phys. Ther..

[25] Trajković N., Kozinc Ž., Smajla D., Šarabon N. (2021). Relationship between ankle strength and range of motion and postural stability during single-leg quiet stance in trained athletes. Sci. Rep..

[26] Francia P., Ferri Marini C., Toni S., Lucertini F., Federici A., Iannone G., Paternostro F., Piccini B. (2022). Lower limb posture and joint mobility in young Soccer players. Italian J. Anat. Embryol..

[27] Ribeiro C.Z.P., Akashi P.M.H., Sacco I.D.C.N., Pedrinelli A. (2003). Relationship between postural changes and injuries of the locomotor system in indoor soccer athletes. Rev. Bras. Med. Esporte.

[28] Hoch M.C., Staton G.S., Medina McKeon J.M., Mattacola C.G., McKeon P.O. (2012). Dorsiflexion and dynamic postural control deficits are present in those with chronic ankle instability. J. Sci. Med. Sport.

[29] Xue X., Ma T., Li Q., Song Y., Hua Y. (2021). Chronic ankle instability is associated with proprioception deficits: A systematic review and meta-analysis. J. Sport Health Sci..

[30] Azuma N., Someya F. (2020). Injury prevention effects of stretching exercise intervention by physical therapists in male high school soccer players. Scand. J. Med. Sci. Sports.

[31] Paterno M.V., Taylor-Haas J.A., Myer G.D., Hewett T.E. (2013). Prevention of overuse sports injuries in the young athlete. Orthop. Clin. N. Am..

[32] Hattori K., Ohta S. (1986). Ankle joint flexibility in college soccer players. J. Hum. Ergol..

[33] Walls R.J., Ross K.A., Fraser E.J., Hodgkins C.W., Smyth N.A., Egan C.J., Calder J., Kennedy J.G. (2016). Football injuries of the ankle: A review of injury mechanisms, diagnosis and management. World J. Orthop..

[34] Kim S.G., Kim W.S. (2018). Effect of Ankle Range of Motion (ROM) and Lower-Extremity Muscle Strength on Static Balance Control Ability in Young Adults: A Regression Analysis. Med. Sci. Monit. Int. Med. J. Exp. Clin. Res..

[35] Beynnon B.D., Murphy D.F., Alosa D.M. (2002). Predictive Factors for Lateral Ankle Sprains: A Literature Review. J. Athl. Train..

[36] Lees A., Asai T., Andersen T.B., Nunome H., Sterzing T. (2010). The biomechanics of kicking in soccer: A review. J. Sports Sci..

[37] Iwamoto Y., Takahashi M., Shinkoda K. (2017). Differences of muscle co-contraction of the ankle joint between young and elderly adults during dynamic postural control at different speeds. J. Physiol. Anthropol..

[38] Kellis E., Katis A. (2007). Biomechanical characteristics and determinants of instep soccer kick. J. Sports Sci. Med..

[39] Ghouchani A., Tabatabai F., Nejad S.K.A., Rahimnejad M. (2010). Analysis of Torques and Forces Applied on Limbs and Joints of Lower Extremities in Free Kick in Football. Procedia Eng..

[40] Youdas J.W., McLean T.J., Krause D.A., Hollman J.H. (2009). Changes in active ankle dorsiflexion range of motion after acute inversion ankle sprain. J. Sport Rehabil..

[41] Corso M. (2018). Developmental changes in the youth athlete: Implications for movement, skills acquisition, performance and injuries. J. Can. Chiropr. Assoc..

[42] Houghton K.M. (2008). Review for the generalist: Evaluation of pediatric foot and ankle pain. Pediatr. Rheumatol. Online J..

[43] Robles-Palazón F.J., Ayala F., Cejudo A., De Ste Croix M., Sainz de Baranda P., Santonja F. (2022). Effects of Age and Maturation on Lower Extremity Range of Motion in Male Youth Soccer Players. J. Strength Cond. Res..

[44] Hogrel J.Y. (2015). Grip strength measured by high precision dynamometry in healthy subjects from 5 to 80 years. BMC Musculoskelet. Disord..

[45] Ploegmakers J.J.W., Hepping A.M., Geertzen J.H.B., Bulstra S.K., Stevens M. (2013). Strength Is Strongly Associated With Height, Weight and Gender in Childhood: A Cross Sectional Study of 2241 Children and Adolescents Providing Reference Values. J. Physiother..

[46] Molenaar H.M., Selles R.W., Zuidam J.M., Willemsen S.P., Stam H.J., Hovius S.E. (2010). Growth diagrams for grip strength in children. Clin. Orthop. Relat. Res..

[47] Strandkvist V., Larsson A., Pauelsen M., Nyberg L., Vikman I., Lindberg A., Gustafsson T., Röijezon U. (2021). Hand grip strength is strongly associated with lower limb strength but only weakly with postural control in community-dwelling older adults. Arch. Gerontol. Geriatr..

[48] Sousa-Santos R., Amaral T.F. (2017). Differences in handgrip strength protocols to identify sarcopenia and frailty—A systematic review. BMC Geriatr..

[49] Benfica P., Aguiar L.T., Brito S., Bernardino L., Teixeira-Salmela L.F., Faria C. (2018). Reference values for muscle strength: A systematic review with a descriptive meta-analysis. Braz. J. Phys. Ther..

[50] Francia P., Toni S., Iannone G., Seghieri G., Piccini B., Vittori A., Santosuosso U., Casalini E., Gulisano M. (2018). Type 1 diabetes, sport practiced, and ankle joint mobility in young patients: What is the relationship?. Pediatr. Diabetes.

[51] Francia P., Bocchi L., Santosuosso U., Iannone G., Vittori A., Toni S. (2021). The effect of different sports specialization on ankle joint mobility of young players. J. Hum. Sport Exerc..

[52] Clarkson H.M. (2013). Musculoskeletal Assessment: Joint Motion and Muscle Testing.

[53] Francia P., Sorelli M., Piccini B., Iannone G., Capirchio L., Toni S., Gulisano M., Bocchi L. (2019). Glycemic Control Maintained over Time and Joint Stiffness in Young Type 1 Patients: What Is the Mathematical Relationship?. J. Diabetes Sci. Technol..

[54] DeLang M.D., Kondratek M., DiPace L.J., Hew-Butler T. (2017). Collegiate male soccer players exhibit between-limb symmetry in body composition muscle strength and range of motion. Int. J. Sports Phys. Ther..

[55] De S., Sengupta P., Maity P., Pal A., Dhara P.C. (2011). Effect of body posture on hand grip strength in adult Bengalee population. J. Exerc. Sci. Physiother..

[56] Mathiowetz V., Weber K., Volland G., Kashman N. (1984). Reliability and validity of grip and pinch strength evaluations. Surg. Am..

[57] Madaleno F.O., Verhagen E., Ferreira T.V., Ribeiro T., Ocarino J.M., Resende R.A. (2021). Normative reference values for handgrip strength, shoulder and ankle range of motion and upper-limb and lower limb stability for 137 youth judokas of both sexes. J. Sci. Med. Sport.

[58] McQuiddy V.A., Scheerer C.R., Lavalley R., McGrath T., Lin L. (2015). Normative Values for Grip and Pinch Strength for 6- to 19-Year-Olds. Arch. Phys. Med. Rehabil..

[59] Cohen J. (1988). Statistical Power Analysis for the Behavioral Sciences.

[60] Boone D.C., Azen S.P. (1979). Normal range of motion of joints in male subjects. J. Bone Joint Surg. Am..

[61] Russell J.A., McEwan I.M., Koutedakis Y., Wyon M.A. (2008). Clinical anatomy and biomechanics of the ankle in dance. J. Dance Med. Sci..

[62] Kumar S., Sharma R., Gulati D., Dhammi I.K., Aggarwal A.N. (2011). Normal range of motion of hip and ankle in Indian population. Acta Orthop. Traumatol. Turc..

[63] Zakas A. (2005). The effect of stretching duration on the lower-extremity flexibility of adolescent soccer players. J. Bodyw. Mov. Ther..

[64] Brown K.A., Patel D.R., Darmawan D. (2017). Participation in Sports in Relation to Adolescent Growth and Development. Transl. Pediatr..

[65] Korff T., Horne S.L., Cullen S.J., Blazevich A.J. (2009). Development of lower limb stiffness and its contribution to maximum vertical jumping power during adolescence. J. Exp. Biol..

[66] Hansen L., Bangsbo J., Twisk J., Klausen K. (1999). Development of muscle strength in relation to training level and testosterone in young male soccer players. J. Appl. Physiol..

[67] Limony Y., Kozieł S., Friger M. (2015). Age of onset of a normally timed pubertal growth spurt affects the final height of children. Pediatr. Res..

[68] Soliman A., De Sanctis V., Elalaily R., Bedair S. (2014). Advances in pubertal growth and factors influencing it: Can we increase pubertal growth?. Indian J. Endocrinol. Metab..

[69] Blazevich A., Waugh C., Korff T., De Ste Croix M., Korff T. (2013). Development of musculoskeletal stiffness. Paediatric Biomechanics and Motor Control Theory and Application.

[70] Croix M. (2007). Advances in paediatric strength assessment: Changing our perspective on strength development. J. Sports Sci. Med..

[71] Vänttinen T., Blomqvist M., Nyman K., Häkkinen K. (2011). Changes in body composition, hormonal status, and physical fitness in 11-, 13-, and 15-year-old Finnish regional youth soccer players during a two-year follow-up. J. Strength Cond. Res..

[72] Viru A., Loko J., Volver A., Laaneots L., Karelson K., Viru M. (1998). Age periods of accelerated improvement of muscle strength, power, speed and endurance in the age interval 6–18 years. Biol. Sport.

[73] Cejudo A., Robles-Palazón F.J., Ayala F., De Ste Croix M., Ortega-Toro E., Santonja-Medina F., Sainz de Baranda P. (2019). Age-related differences in flexibility in soccer players 8–19 years old. PeerJ.

[74] Wind A.E., Takken T., Helders P.J.M., Engelbert R.H.H. (2010). Is grip strength a predictor for total muscle strength in healthy children, adolescents, and young adults?. Eur. J. Pediatr..

